# A Cluster of *Plasmodium ovale* Infections in Belgian Military Personnel after Deployment in Kindu, Democratic Republic of Congo: A Retrospective Study

**DOI:** 10.3390/tropicalmed6030125

**Published:** 2021-07-08

**Authors:** Diana Isabela Costescu Strachinaru, An Wauters, Marjan Van Esbroeck, Mihai Strachinaru, Peter Vanbrabant, Patrick Soentjens

**Affiliations:** 1 Center for Infectious Diseases, Queen Astrid Military Hospital, 1120 Brussels, Belgium; peter.vanbrabant@mil.be (P.V.); psoentjens@itg.be (P.S.); 2 Department of Clinical Sciences, Institute of Tropical Medicine, 2000 Antwerp, Belgium; wauters_an@hotmail.com (A.W.); mvesbroeck@itg.be (M.V.E.); 3 Department of Cardiology, Erasmus Medical Center, 3000 CA Rotterdam, The Netherlands; m.strachinaru@erasmusmc.nl; 4 General Internal Medicine, University Hospitals Leuven, 3000 Leuven, Belgium

**Keywords:** malaria, non-falciparum malaria, neglected disease, *Plasmodium ovale*, military

## Abstract

*Plasmodium ovale* malaria is often neglected due to its less severe course compared to *Plasmodium falciparum*. In 2011–2012, Belgian Armed Forces identified a cluster of *P. ovale* cases among military personnel after deployment in the Democratic Republic of Congo (DRC). In this retrospective, monocentric, observational study, clinical and biological features of soldiers diagnosed with *P. ovale* after deployment in DRC were reviewed. Species diagnosis was based on polymerase chain reaction (PCR) and/or thick blood smear. Medical records of 149 soldiers screened at the Queen Astrid Military Hospital after deployment were reviewed. Eight cases (seven *P. ovale* infections and one *P. ovale—falciparum* coinfection) were identified. All had positive thick smears, and seven were confirmed by PCR. Chemoprophylaxis was mefloquine in all subjects. Median time of disease onset was 101 days after return from the endemic region. Median delay between return and diagnosis was 103 days. All *P. ovale* bouts were uncomplicated. None had relapses after primaquine treatment. This military cohort highlights a hotspot of *P. ovale* in Eastern DRC. Non-specific symptoms, the less severe presentation, the lack of sensitive parasitological tools in the field and long delays between infection and symptoms probably lead to underestimation of *P. ovale* cases.

## 1. Introduction

Malaria is the most common cause of fever in returning travelers and migrants from endemic regions [1,2]. Military personnel deployed in these regions are also at risk [3,4]. *Plasmodium ovale* (*P. ovale)* is endemic to areas in Central and West Africa and in Southeast Asia [5]. In populations with endemic circulation, fever occurs when parasitemia exceeds 800 parasites/µL [5]. *P. ovale* malaria usually has a less severe clinical course when compared to *Plasmodium falciparum* *(P. falciparum)* and deadly cases are rare; therefore, it is often neglected. A systematic review of *P. ovale* malaria published in 2017, which included 33 research studies, describes only 18 cases of relapse of *P. ovale*, 22 serious cases and five related deaths [5]. There are relatively few imported cases outside of endemic regions and the true burden of *P. ovale* disease among civilian travelers or deployed troops remains little known. Recently, several countries reported cases of *P. ovale* infections among returning military personnel [6,7,8,9]. According to the GeoSentinel surveillance network, the average time between the return and clinical presentation for *P. ovale* is 82 days, in contrast to 17.5 days for *P. falciparum* [10]. The long latency after a stay in an endemic zone as well as low specificity of clinical signs and low parasitemia make *P. ovale* diagnosis challenging.

From August 2011 to April 2012, a detachment of the Belgian Armed Forces was deployed in Kindu, Eastern Democratic Republic of Congo (DRC), in a region with a high estimated malaria burden, with many perennial water areas and a high density of well-adapted vectors [11]. The most up-to-date epidemiological data concerning the DRC at that time come from a study conducted in 2007, which found *P. falciparum* to be the most prevalent species, either as monoinfection (90.4%; 95% C.I. 88.8–92.1) or as coinfection with *P. malariae* (4.9%; 95% confidence interval, 3.7–5.9), *P. ovale* (0.6%; 95% confidence interval, 0.1–0.9), or all three species (0.1%; 95% confidence interval 0–0.3) [12]. *P. ovale* parasitemia was found to be rare overall, as were monoinfections with *P. ovale* (0.1%; 95% confidence interval 0.01–0.2) [12]. During the deployment period, 14 suspected episodes of malaria in members of this detachment were attributed to *P. falciparum*. The diagnoses were made locally in symptomatic individuals using microscopy and/or rapid diagnostic tests (RDTs) and cases were treated with either artemether/lumefantrine (AL) or atovaquone/proguanil (AV + PG). Following this alert, military personnel belonging to the concerned detachment and any other military personnel having been deployed in DRC in the same period and having had symptoms during deployment were targeted for active malaria screening after returning to Belgium: symptomatic patients underwent RDT, thick smear and/or polymerase chain reaction (PCR) and serological tests, and asymptomatic patients were only screened with serological tests. The goal of this study was to identify in this cohort all *P. ovale* infections, to describe their course and to analyze the demographic, epidemiological, clinical and laboratory data of the infected personnel.

## 2. Materials and Methods

### 2.1. Study Design and Population

This was a retrospective, monocentric, observational study, in which the medical files of all subjects from the targeted cohort with at least one post-mission appointment in the Queen Astrid Military Hospital in Brussels (QAMH) were reviewed. The following data were registered: demographic information (age, sex, country of birth), duration of the mission, self-declared compliance to the use of mosquito avoidance measures as well as self-declared compliance to anti-malaria chemoprophylaxis and type of chemoprophylaxis used, previous stays in malaria endemic regions, results of previous malaria serological tests when available, clinical presentation during and after the mission, type of malaria infection, interval between the date of return to Belgium and onset of symptoms, interval between the date of return to Belgium and the diagnosis, diagnostic tests used and the treatment regimen received. All the patients had a complete treatment course, including antirelapse therapy with primaquine (PQ) 30 mg (base) daily for 14 days. Glucose-6-phospatedehydrogenase (G6PD) level, occurrence of adverse events and outcome were recorded.

#### Inclusion Criteria

We included all patients having either:

- a positive thick and thin smear showing *P. ovale* parasites and/or;
- a positive polymerase chain reaction (PCR) for *P. ovale*.

### 2.2. Definitions and Microbiology

The case definition of a confirmed malaria case was onset of symptoms associated with the presence of *Plasmodium* on thick blood smears and/or positivity of an RDT (antigenic tests: DiaMed OptiMal-IT^®^ rapid malaria test) and/or positive PCR. Species identification was based on microscopic examination (thick and thin smear) and/or PCR. For the PCR tests, DNA was extracted from 200 µL of EDTA whole blood samples with the Qiagen DNA mini kit (Qiagen Benelux, Venlo, The Netherlands). PCR was performed by real-time PCR with four Plasmodium species-specific probes. Two duplex reactions were run in parallel, one detecting *P. falciparum*/*P. vivax* and one detecting *P. ovale*/*P. malariae*. PCRs were performed on a SmartCycler II (Cepheid Benelux, Bouwel, Belgium) [13]. Detection of malaria antibodies was performed by an indirect immunofluorescence assay using antigens of *P. falciparum*, *P. ovale* and *P. malariae* [14].

### 2.3. Statistics 

Discrete variables are represented as percentage. Continuous normally distributed variables are represented as mean ± standard deviation. In case of abnormal distribution, data are presented as median and interquartile range. Statistical analyses were conducted in SPSS 21 (IBM, Armonk, NY, USA, 2012). A *p* value of <0.05 was considered significant.

## 3. Results

**Figure 1:**
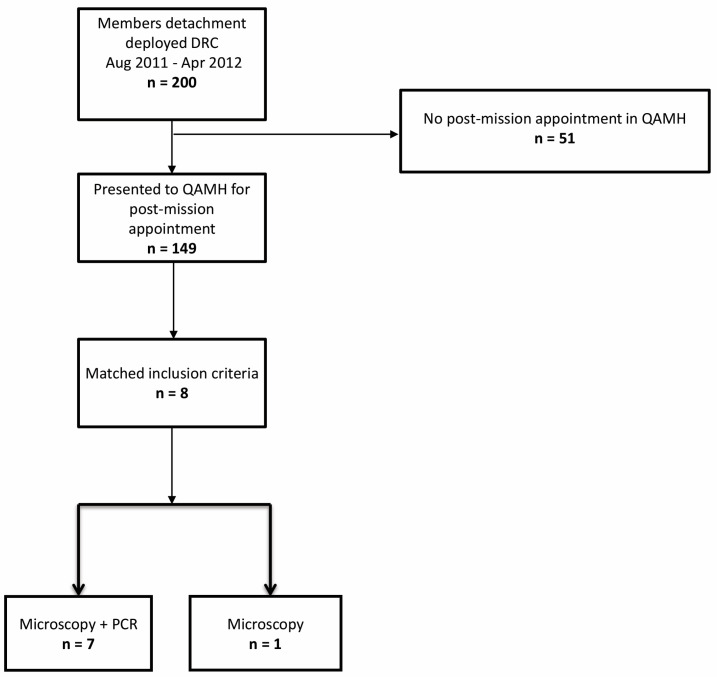
Inclusion flowchart. DRC: Democratic Republic of Congo, QAMH: Queen Astrid Military Hospital.

The follow-up ran from October 2011 to December 2015. Active surveillance (doctor’s appointments and subsequent phone calls and mailing) was performed to obtain information on the outcome. Figure 1 presents the patient selection algorithm.

In order to assess the malaria chemoprophylaxis against *P. ovale,* we analyzed the entire cohort screened at the QAMH (149 subjects). The mean duration of deployment was 85 ± 32 days (range: 7–191). Data concerning the type of chemoprophylaxis were available for all subjects. Prophylaxis consisted of mefloquine (MQ) (n = 94, 63.08%), doxycycline (DOXY) (n = 16, 10.73%) or AV + PG (n = 4, 2.68%). The remaining 35 subjects (23.48%) had to switch from their initial treatment to another molecule due to side effects. Of these, 28 (18.79%) had to switch to MQ. Compliance with chemoprophylaxis was reported by 114/149 subjects (76.51%). Because of the ratio between the number of events on one side and the number of subjects receiving other medication than MQ, medication could not be entered into a regression analysis.

**Figure 2:**
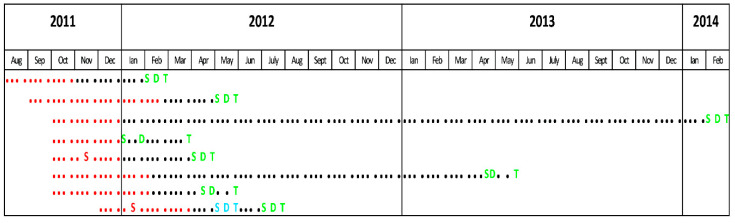
Timeline. Red dots: mission duration. Black dots: time between the return from the endemic region and the events related to *P. ovale* infection. S: symptoms during mission. S: symptoms due to a *P. falciparum* attack. **S**: symptoms due to a *P. ovale* attack. D: *P. falciparum* diagnosis. D: *P. ovale* diagnosis. T: *P. falciparum* treatment. T: *P. ovale* treatment (including primaquine eradication therapy).

Eight patients met the inclusion criteria for our study (seven cases of *P. ovale* infection and one *P. ovale—falciparum* mixed infection were diagnosed during the active screening period). All subjects had positive thick and thin smears. Seven cases (87.5%) were confirmed by PCR (in one case PCR was not performed). All infected subjects were men. Median age was 36 years (IQR = 30–45, range: 23–50). All patients were born in Belgium. All had been deployed in the same area. The median duration of deployment was 88 days (IQR = 61–110, range: 61–148). Five (62.5%) had previous deployments in one or several malaria endemic regions. No data regarding previous personal voyages in malaria endemic regions were available. For two subjects (25%), malaria serology had previously been performed: tests were negative for *P. ovale*, *P. falciparum* and *P. malariae*. No testing for *P. vivax* was available. None of the subjects had previously been treated for malaria. Data concerning the use of mandatory mosquito avoidance measures (use of N, N-diethyl-meta-toluamide (DEET), impregnated clothes and mosquito nets) were available for seven (87.5%) subjects: six (75%) reported compliance. All subjects used MQ as chemoprophylaxis. Correct use of chemoprophylaxis was reported by 6/8 subjects (75%). Four subjects (50%) presented some symptoms and only two (25%) had fever during deployment; none received specific malaria treatment. All eight subjects presented symptoms after their return to Belgium. The most common symptoms were fever and chills, encountered in seven patients (87.5%), followed by headache—six patients (75%), cough, dyspnea and diarrhea—three patients (37.5%) each and abdominal pain—one patient (12.5%). Symptom onset median time was 101 (IQR = 47–334) days after return (range: 29–775) and the median delay between the return and the diagnostic was 103 (IQR = 62–339) days (range: 30–789). The timelines between the periods spent in the malaria endemic region, the apparition of symptoms, the malaria diagnosis and treatment are presented in Figure 2. Only two patients (25%) presented splenomegaly at examination. Median hemoglobin, platelet count and parasitemia was 12.8 g/dL, 122.000/mm^3^ and 458 parasites/µL, respectively. The results of RDTs performed on the same day as the microscopic examination were known in six patients (75%); all were negative. All attacks of *P. ovale* malaria were considered uncomplicated. Admission to a hospital was necessary in three (37.5%) patients, one of which was coinfected with *P. falciparum*. The initial treatment of *P. ovale* infections was chloroquine (CQ) in five patients (%), AV + PG in two patients (including the case of *P. falciparum* coinfection) and AL in one case. None had G6PD deficiency. All the patients received eradication treatment with PQ. None had relapses after PQ treatment.

## 4. Discussion

This study presents a cluster of eight *P. ovale* infections in a military population deployed in DRC, revealed through active surveillance that allowed the detection of rare events such as non-falciparum malaria. This cluster was of concern for the military medical authorities for several reasons: the risk of incapacitation of infected soldiers in the field, the availability of only insensitive diagnostic tools such as RDTs and the need for an eradication treatment (radical cure) with PQ to avoid subsequent relapses related to hypnozoites. Despite a relatively high percentage of self-reported use of preventive measures, long periods in a highly endemic region with many permanent water areas and high exposure to vectors resulted nevertheless in malaria infections. The majority of the subjects in the cohort screened at QAMH had used MQ and 76.5% reported correct use of chemoprophylaxis. Therefore, it was impossible to statistically evaluate the role of MQ as protection against *P. ovale* infection. MQ, a methanol-quinoline drug that works as a blood schizonticide, was developed by the US military in the 1970s, in response to the increase in CQ resistance in *P. falciparum* in Southeast Asia [15]. It has been used in the military since the mid-eighties and is still used as the first-choice malaria chemoprophylaxis in some countries, such as Peru and Indonesia [7,8]. While there is abundant literature reviewing the drug’s risks and adverse events in civilian travelers as well as in the military, there are few data concerning MQ efficacy in preventing *P. ovale* infections [16]. This cluster occurred at a time when first-choice malaria chemoprophylaxis regimen in Belgian military was being switched from MQ to DOXY because of MQ’s risks. DOXY, the current first-choice malaria chemoprophylaxis regimen in French, Belgian and the US military appears to be less effective against *P. ovale*. French military personnel experienced an increase in *P. ovale* infections in troops deployed to the Ivory Coast between 2002 and 2007, coinciding with the change of chemoprophylaxis from CQ with PG to DOXY. This phenomenon was interpreted as potential proof of the lower effectiveness of DOXY on *P. ovale* than on *P. falciparum* [6]. To date, there is no well-tolerated chemoprophylaxis with a validated efficacy against *P. malariae, P. vivax* and *P. ovale* attacks [6]. MQ’s risks and adverse events combined with a potentially lower effectiveness of DOXY against non-falciparum malaria raises practical concerns for malaria chemoprophylaxis in travelers and military deployed in regions endemic for *P. falciparum* and *Plasmodium* non-falciparum. Further studies are needed to assess the efficacy of the current prophylactic regimens against the non-falciparum malaria parasites. Tafenoquine (TQ), a new antimalarial drug with a long half-life, was recently approved by the US Food and Drug administration for malaria prophylaxis [17]. However, it can only be used in patients with normal G6PD activity and is contraindicated in children, pregnant women and breastfeeding women with infants having a G6PD deficiency or unknown G6PD status [17].

One of the interests of this series derives from the long-term medical monitoring by the Belgian military health department. While the average time between the return from a malaria endemic region and clinical presentation is 82 days for *P. ovale*, some authors reported very late *P. ovale* infections in travelers or returning military personnel: up to 45 months in a French patient, between 2 and 53 months after return from West Africa in 16 Spanish travelers, 47 and 69 months in two French military men returning from the Ivory Coast and up to 11 months after return from the Central African Republic in Peruvian peacekeepers [8,10,18,19,20]. In this cohort, the longest delay between the return from DRC and disease onset was 25 months. This patient had no previous or subsequent voyages in a malaria endemic zone and presented with fever, chills and asthenia 775 days after return. The diagnosis was only made 789 days after the return, when the microscopy, PCR and serology were positive for *P. ovale*. He received CQ followed by PQ, without subsequent attacks. This case re-emphasizes the possibility of long delays between the return from an endemic area and the occurrence of a *P. ovale* attack and highlights the importance of a complete travel history in patients presenting with unexplained fever, even years after a stay in the tropics.

The poor sensitivity of the parasitological diagnostic tools to detect *P. ovale,* especially in cases with low parasitemia, further hinders the diagnosis [21,22,23,24]. *P*. *ovale* infection is difficult to diagnose microscopically in the field. Because of the generally low parasitemia in patients and because the morphology of *P*. *ovale* resembles that of *Plasmodium vivax,* this technique requires highly trained and experienced personnel, which are not always available in the field. Moreover, microscopy itself is not always readily available. Therefore, diagnosis during deployment sometimes relies only on RDTs. Currently available RDTs have low performance in detecting *P. ovale* infections, especially in cases with low parasitemia [24]. In our series, none of the RDTs were positive, probably due to the patients’ low parasitemia. To this day, there is no *P. ovale*-specific RDT available on the market, and RDTs can therefore only identify *Plasmodium* up to the non-falciparum level. While molecular detection methods such as PCR represent a real step forward in confirming the diagnosis, they are not routinely available and, to our knowledge, they have yet to be used as a diagnostic tool in the field.

Furthermore, *P. ovale* is believed to be responsible for asymptomatic cases. Doderer-Lang et al. reported high rates of antibodies against *P. ovale* and *P. malariae* in asymptomatic blood donors from Benin [25]. Asymptomatic cases, the less severe clinical presentation in the case of symptoms, the lack of sensitive parasitological tools in the field and long delays between infection and symptoms probably lead to underdiagnosis of *P. ovale* cases.

*P. ovale* infections need ensuring the eradication of liver hypnozoites to prevent relapses (radical cure) in addition to treating the erythrocytic asexual forms that cause symptoms [26,27]. The only alternative currently available in Belgium is PQ. TQ allows a user-friendly single dose that provides equivalent efficacy to 14 days of PQ in achieving radical cure of Plasmodium non-falciparum [17]; however, it has yet to be registered in Belgium.

### Limitations

The relatively small number of patients and the fact that the cohort consists only of young adult males means that this group may not be representative for travelers or for populations living in malaria endemic settings. Another limitation is the lack of previous serological testing in the group. Results of previous malaria serology were only available for two subjects; both were negative. All included soldiers were born in non-endemic areas, but 62.5% had previous deployments in endemic regions and no data regarding previous personal voyages were available. Therefore, we cannot affirm with certainty that our cohort represents a non-immune population. Another important limitation is the self-reporting nature of compliance with the use of the mosquito avoidance measures and with anti-malaria chemoprophylaxis. In this study, the compliance was measured based on a self-administered questionnaire filled during the first medical visit after the mission. This may have overestimated the rate of compliance, as previously shown for anti-viral therapy [28] and malaria chemoprophylaxis [29].

However, despite the limitations, we believe that this short report adds another piece to the puzzle of imported non-falciparum malaria. A recent study shows that *P. ovale* remains broadly prevalent in the DRC (prevalence estimate 0.8%; 95% confidence interval, 0.59–0.98%) [30]. We therefore want to raise clinicians’ awareness of the challenges posed by this neglected disease in both endemic and non-endemic settings.

## 5. Conclusions

This military cohort reveals a hotspot of *P. ovale* in Eastern DRC. Late onset of symptoms can lead to delayed patient presentation and delayed diagnosis or misdiagnosis. The non-specific symptoms and the poor sensitivity of parasitological diagnostic tools further hinder *P. ovale* diagnosis. This probably leads to an underestimation of *P. ovale* cases in clinical practice.

## Data Availability

The data presented in this study are available on request from the corresponding author. The data are not publicly available due to patient privacy.
